# Association between general self-efficacy and health literacy among stroke survivors 1-year post-discharge: a cross-sectional study

**DOI:** 10.1038/s41598-024-57738-z

**Published:** 2024-03-27

**Authors:** Andrea Hess Engström, Maria Flink, Sebastian Lindblom, Lena von Koch, Charlotte Ytterberg

**Affiliations:** 1 Department of Neurobiology, Care Sciences and Society, Karolinska Institute, Stockholm, Sweden; 2 Women’s Health and Allied Health Professionals Theme, Karolinska University Hospital, Stockholm, Sweden; 3 Theme of Heart and Vascular and Neuro, Karolinska University Hospital, Stockholm, Sweden

**Keywords:** Health care, Neurology

## Abstract

Stroke may affect physical functioning, cognition, and mental and social aspects of one’s life. Health literacy and self-efficacy are associated with positive health outcomes and are important factors for managing the diverse consequences of a stroke. However, there is very little literature on the association between health literacy and self-efficacy. This study aimed to investigate the association between health literacy and self-efficacy among stroke survivors 1 year after discharge from hospital. Participants in this cross-sectional study were patients diagnosed with a stroke, mainly a mild stroke, who were referred to rehabilitation in primary care after discharge from hospital in Sweden. Data was collected using questionnaires, performance-based tests, and medical records. Ordinal logistic regression was used to analyze the association between general self-efficacy and health literacy in adjusted models. The analysis revealed that higher levels of general self-efficacy and higher levels of performing activities of daily living were associated with higher levels of health literacy. Stroke survivors with higher general self-efficacy also report higher health literacy 1-year post-discharge from hospital. Future studies should focus on the pathways by which health literacy and general self-efficacy work among stroke survivors and in populations with low health literacy, severe stroke or significant cognitive impairments.

## Introduction

Stroke is the second leading cause of death worldwide and the third leading cause of death and disability combined^1^. In Europe, an incidence of 1.12 million cases of stroke was estimated in 2017, and a 30-years projection has predicted an increase in the numbers of stroke events and stroke survivors^2^. Stroke survivors may experience a range of impairments related to motor or cognitive functions, or a combination of both. It has been observed that physical impairments can be linked to a decline in overall cognition, executive function, and memory^3^.

There is a cumulative risk of stroke recurrence of 26% during the 5 years following an initial stroke^4^. Therefore, secondary stroke prevention is necessary for stroke survivors to reduce the risk of recurrence. Secondary stroke prevention comprises surgical and pharmacological interventions, but behavioral changes may also be required, such as smoking cessation, reduction in alcohol consumption, participation in exercise, and dietary changes to reduce lifestyle-related risk^5,6^. These behavioral changes require substantial knowledge, motivation, and competencies^7^.

Health literacy is the knowledge, motivation, and competencies that determine a person’s ability to access, understand, appraise, and apply health information in order to promote and maintain good health and make appropriate health decisions^8^. Health literacy is associated with improved health behavior and health status among patients with cardiovascular disease^9^. Among stroke survivors, greater health literacy is associated with better function^10^, and better overall health^11^. In a previous study, higher levels of health literacy were associated with positive outcomes related to symptoms of depression, walking ability, perceived stroke recovery, and perceived participation^12^. Furthermore, stroke survivors with inadequate health literacy had poorer retention of health education and recalled only half of the stroke educational material they had received^13^. This may complicate the undertaking of measures for secondary stroke prevention. Despite the importance of health literacy, there are few studies that specifically target health literacy in stroke survivors.

In addition to health literacy, self-efficacy, i.e., the belief that one can successfully perform a behavior required to produce certain outcomes^14^, is important for stroke survivors. General self-efficacy can be conceptualized as a global and broad belief in one’s ability to deal effectively with new and stressful situations and can be assessed as a unidimensional and universal construct^15^, which may be necessary to manage the diverse consequences of a stroke. For example, it has been reported that higher levels of general self-efficacy are associated with lower levels of depression among stroke survivors^16^, whereas depression and older age are associated with low levels of self-care self-efficacy^17^. Improved self-efficacy can also have a positive impact on health decisions and contribute to promoting changes in health behavior^18^. For example, high levels of self-care self-efficacy are associated with increased quality of life and lower levels of depression^19^, whereas high levels of fall-related self-efficacy are associated with better physical functioning and improved performance of activities of daily living among stroke survivors^19^.

Hence, both health literacy and self-efficacy are independently associated with health outcomes for stroke survivors. The association between self-efficacy and health literacy has previously been investigated in other populations, but mainly focusing on how health literacy affects self-efficacy^20,21^. Only one study has investigated this association in stroke survivors, and showed that using a 5-min multidisciplinary intervention to increase health literacy among stroke survivors after discharge from hospital improved patients’ self-efficacy in stroke symptom recognition and knowledge about stroke^20^. Despite the importance of health literacy and self-efficacy for managing the diverse consequences of a stroke, there is little literature on the association between health literacy and self-efficacy among stroke survivors. Stroke may affect physical functioning, cognition, and the mental and social aspects of one’s life^3,22–24^, which can affect the management of stroke consequences. As health literacy can be promoted through interventions delivered by health professionals^25^, and as high self-efficacy is a factor that positively influences stroke recovery, it is relevant to the field to further study the relationship between general self-efficacy and health literacy among stroke survivors^26^.

### Aim

This study aimed to investigate the association between general self-efficacy and health literacy among stroke survivors 1 year after discharge from hospital.

## Methods

### Study participants

In Stockholm, Sweden, patients who have suffered a stroke are admitted for acute hospital care and rehabilitation at a specialized stroke unit. Participants were patients with a stroke diagnosis who had been referred to neurorehabilitation teams in primary care after discharge from one of two study hospitals and who were included in a longitudinal study with baseline data and follow-ups at 3 and 12 months, carried out between 2016 and 2018. All eligible patients received oral and written information about the study and were included after a signed informed consent form was obtained. Recruitment was carried out by members of the rehabilitation teams working at participating hospitals. Detailed information on selection and recruitment has been described previously^27,28^. Those eligible to participate in the present study were participants who at 12 months had completed the European Health Literacy Survey Questionnaire and General Self-Efficacy Questionnaire.

The study was approved by the Regional Ethics Committee in Stockholm (registration number 2015/1923-31/2) and is reported according to the STROBE guidelines. All research was performed in accordance with the Declaration of Helsinki.

### Data collection

We collected data using questionnaires, performance-based tests, and medical records.

Sociodemographic data was retrieved from medical records and included, age, sex, educational level (elementary, secondary, university/college), working status (yes/no), and cohabiting status (yes/no).

The Modified Rankin Scale (mRS) was used to present the severity of disability 1 year after the stroke. The instrument uses a 6-point scale ranging from 0 to 5, and can be categorized as no symptoms (0), mild disability (1–2), moderate disability (3–4), and severe disability (5)^29^.

The outcome variable, health literacy, was assessed using the Swedish version of the European Health Literacy Survey Questionnaire (HLS)^30^.The questionnaire consists of 16 items with five alternatives per item (very easy, easy, difficult, very difficult, and I don’t know), and comprises different dimensions of health literacy: the ability to access, understand, process, and apply health information^31^. Total scores range from 0 to 16, and are categorized as follows: ≤ 8 = inadequate health literacy, 9–12 = problematic, and ≥ 13 = sufficient^31^ (Supplementary information).

#### Independent variables

The *General self-efficacy scale* (GSES) was used to assess general perceived self-efficacy. This form consists of 10 items and responses are given on a 4-point scale. The total score ranges between 10 and 40, with higher scores indicating greater perceived self-efficacy.

The *Barthel Index* (BI) was used to assess the need for assistance in performing personal activities of daily living^32,33^. The questionnaire consists of 10 items about personal care and mobility. The scores range between 0 and 100, with higher scores indicating greater independence^33^.

The *Montreal Cognitive Assessment* (MoCA) was used to assess mild cognitive impairment. The MoCA is divided into different domains: attention and concentration, executive function, memory, linguistic ability, visuo-constructive abilities, abstract thinking, numeracy, and orientation. The highest score is 30 and a cutoff of 26 points has been recommended for patients at 1 year after a stroke, where 26 points and above is considered to indicate no cognitive impairment^34^.

The *Fatigue Severity Scale* (FSS) was used to assess fatigue after a stroke. This instrument consists of nine statements ranging from 1 to 7 (disagree–agree)^35^. Higher scores indicate greater fatigue and a mean score of ≥ 4 points is commonly used to classify fatigue after stroke^36^.

The subscale Depression from the *Hospital Anxiety and Depression Scale* (HADS-D) was used to assess symptoms of depression. The subscale comprises seven items and the scores range from 0 to 3. The total possible score for this subscale is 21, with higher scores indicating greater levels of depression symptoms. A score of ≥ 4 has been previously suggested as a cutoff for depression symptoms after stroke^37^.

The *Stroke Impact Scale* (SIS)*, domain participation* was used to assess the perceived impact of stroke on participation in activities such as work, social activities, recreation, role as a family member or friend, religious or spiritual activities, ability to control one’s own life, and ability to help others^38^. The score ranges from 0 to 100, with higher scores indicating lower perceived impact.

### Statistical analysis

Descriptive statistics were used to describe socio-demographic information, stroke severity, and the variables used in the statistical models.

Ordinal logistic regression was used to identify the association between general self-efficacy and the outcome variable health literacy and carried out according to the following models: Model A (*GSES, Age, Sex, BI*), Model B (*GSES, Age, Sex, BI*, *Education level*), Model C (*GSES, Age, Sex, BI, Education level, MoCA*), Model D (*GSES, Age, Sex, BI, Education level, MoCA, FSS*), Model E (*GSES, Age, Sex, BI, Education level, MoCA, FSS, HADS-D*)*,* and Model F (*GSES, Age, Sex, BI, Education level, MoCA, FSS, HADS-D, SIS participation*).

The outcome variable, health literacy, was analyzed as a categorical variable (inadequate, problematic, sufficient) in the regression models. The variables GSES and age were analyzed as continuous variables. The other variables were analyzed as dichotomous variables: sex (male/female), education level (elementary and secondary/university or college), BI (0–95, dependent/95–100, independent), MoCA (up to 25/26–30), and SIS participation (0–84/85–100), and FSS (fatigue ≥ 4 points/no fatigue 0–3 points).

The accuracy of the statistically significant predictions was analyzed using receiver-operating-characteristic (ROC) curve analysis and reported as sensitivity, specificity, area under the curve (AUC), and 95% confidence interval. Health literacy was analyzed in these models as two dichotomous variables: inadequate and problematic health literacy versus sufficient health literacy, and inadequate health literacy versus problematic and sufficient health literacy.

Results were considered statistically significant if *p* < 0.05. All analyses were carried out using IBM SPSS Statistics, version 28.0.1.1.

## Results

The participants had a mean age of 72 years (SD = 12) and the majority were male (64%). Most of the participants had at least a high-school education (71%) and reported no or mild disability after the stroke (81%) (Table 1).

**Table 1:** Participants’ characteristics 1 year after discharge from hospital.

Participants’ characteristics (n = 108)	
Age at inclusion [mean (SD)]	72 (12)
Sex [n (%)]	
Male	69 (64)
Female	39 (36)
Education level [n (%)]	
Elementary	31 (29)
Secondary	25 (23)
University/College	52 (48)
Working [n (%)]	30 (28)
Cohabitation with partner [n (%)]	74 (69)
MRS [n (%)]*	
No symptoms (0)	26 (24)
Mild disability (1–2)	61 (57)
Moderate disability (3–4)	20 (19)
Severe disability (5)	0 (0)

*Missing n = 1.

Health literacy was sufficient among almost two thirds of the sample (62%). General self-efficacy was above 30 points on the 0–40-point scale among 66% of the participants. Other characteristics of participants—such as cognitive impairment, fatigue, depression symptoms, and the perceived impact of stroke on participation in activities—are presented in Table 2.

**Table 2:** Descriptive information about the variables used in the statistical models.

Variable	N (%) and median (IQR)
Health literacy scale	
Sufficient	67 (62)
Problematic	31 (29)
Inadequate	10 (9)
Median (IQR)	14 (11–16)
General self-efficacy (GSES)	
Mean (SD)	30.5 (6.6)
Barthel index	
Independent = 100	83 (77)
Dependent = less than 100	25 (23)
Median (IQR)	100 (100–100)
Montreal cognitive assessment*	
More than 26	52 (55)
1–25	43 (45)
Median (IQR)	26 (23–28)
HADS-depression	
0–3	77 (71)
At least 4	31 (29)
Median (IQR)	2(1–4)
Fatigue severity scale**	
< *3.99* = *no fatigue*	59 (55)
≥ *4* = *fatigue*	48 (45)
Median (IQR)	3.2 (2–4.4)
SIS participation	
85–100	59 (55)
0–84	49 (45)
Median (IQR)	88.9 (64–100)

*Missing n = 12, **Missing n = 1.

General self-efficacy was significantly associated with health literacy in all regression models, regardless of adjustments (Table 3). The only additional variable that remained statistically significant in all regression models was the need for assistance in performing personal activities of daily living assessed using the BI. Higher levels of general self-efficacy and higher levels of independence in performing activities of daily living were associated with greater levels of health literacy.

**Table 3:** Ordinal logistic regression models of associations between general self-efficacy and health literacy.

Independent factors*	Odds ratio	95% CI	*p*-value
Model A (n = 108)			
GSES	**1.13**	**1.06–1.20**	** < 0.001**
Age	1	0.96–1.04	0.99
Sex	0.72	0.30–1.75	0.47
BI	**6.67**	**2.45–18.19**	** < 0.001**
Model B (n = 108)			
GSES	**1.14**	**1.06–1.22**	** < 0.001**
Age	0.99	0.96–1.03	0.83
Sex	0.75	0.30–1.83	0.52
BI	**6.4**	**2.35–17.39**	** < 0.001**
Education level	1.63	0.66–4.04	0.29
Model C (n = 95)			
GSES	**1.13**	**1.05–1.21**	** < 0.001**
Age	1	0.96–1.04	0.9
Sex	0.75	0.28–2.00	0.57
BI	**5.43**	**1.75–16.83**	**0.003**
Education level	1.63	0.63–4.24	0.31
MoCA	1.56	0.59–4.12	0.37
Model D (n = 94)			
Self-efficacy	**1.11**	**1.03–1.19**	**0.01**
Age	1	0.97–1.05	0.69
Sex	0.66	0.24–1.82	0.43
BI	**5.93**	**1.86–18.80**	**0.003**
Education level	1.65	0.63–4.32	0.3
MoCA	1.5	0.55–4.11	0.43
FSS	0.56	0.20–1.63	0.29
Model E (n = 94)			
GSES	**1.1**	**1.01–1.19**	**0.02**
Age	1	0.97–1.05	0.74
Sex	0.66	0.24–1.81	0.41
BI	**5.91**	**1.84–18.88**	**0.003**
Education level	1.65	0.63–4.32	0.31
MoCA	1.3	0.50–3.87	0.53
FSS	0.62	0.21–1.85	0.39
HADS-D	0.68	0.22–2.07	0.5
Model F (n = 94)			
GSES	**1.09**	**1.01–1.19**	**0.03**
Age	1.01	0.96–1.05	0.77
Sex	0.61	0.21–1.77	0.36
BI	**5.14**	**1.45–18.21**	**0.01**
Education level	1.57	0.60–4.14	0.36
MoCA	1.34	0.48–3.75	0.57
FSS	0.63	0.21–1.88	0.4
HADS-D	0.72	0.23–2.22	0.57
SIS participation	1.34	0.43–4.22	0.61

*GSES, General Self-Efficacy Scale; BI, Barthel Index; FSS, Fatigue Severity Scale; MoCA, Montreal Cognitive Assessment; HADS-D: Hospital and Anxiety Scale, Depression; SIS participation, Stroke Impact Scale 3.0 (SIS), domain participation.

Significance values are bold.

### Accuracy of the predictions

**Figure 1:**
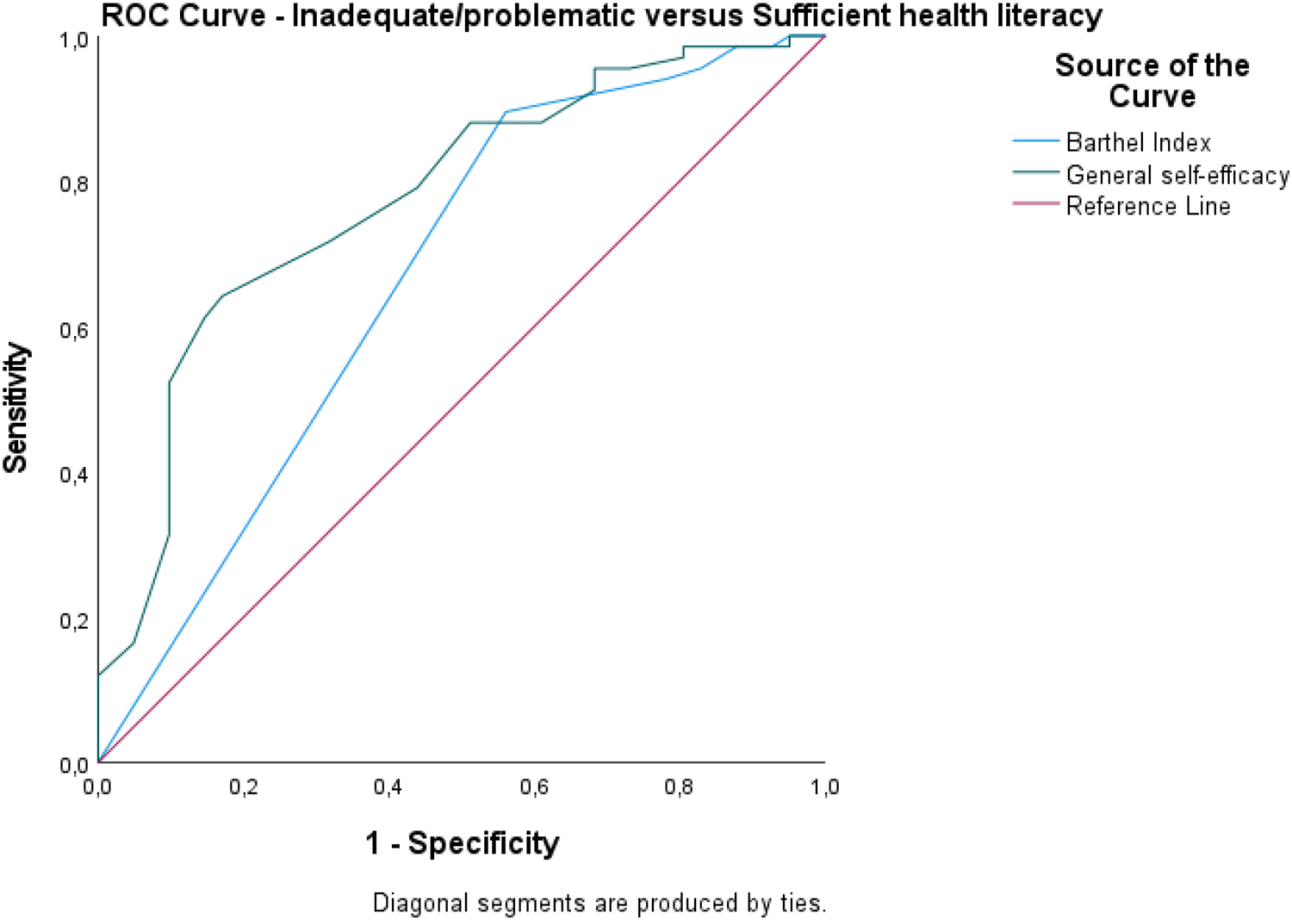
Specificity and sensitivity based on dichotomous variable health literacy (inadequate/problematic vs. sufficient).

The ROC curve for the predictions based on health literacy (inadequate/problematic vs. sufficient health literacy) is presented in Fig. 1. The area under the curve for general self-efficacy was 0.78 (CI 0.69–0.87), and for the BI it was 0.67 (CI 0.56–0.78). For general self-efficacy, the model had 1.0 sensitivity and 0.95 specificity at a 14.50 cutoff. At an 82.50 cutoff from the BI, the model presented 0.99 sensitivity and 0.88 specificity Fig. 1.

**Figure 2:**
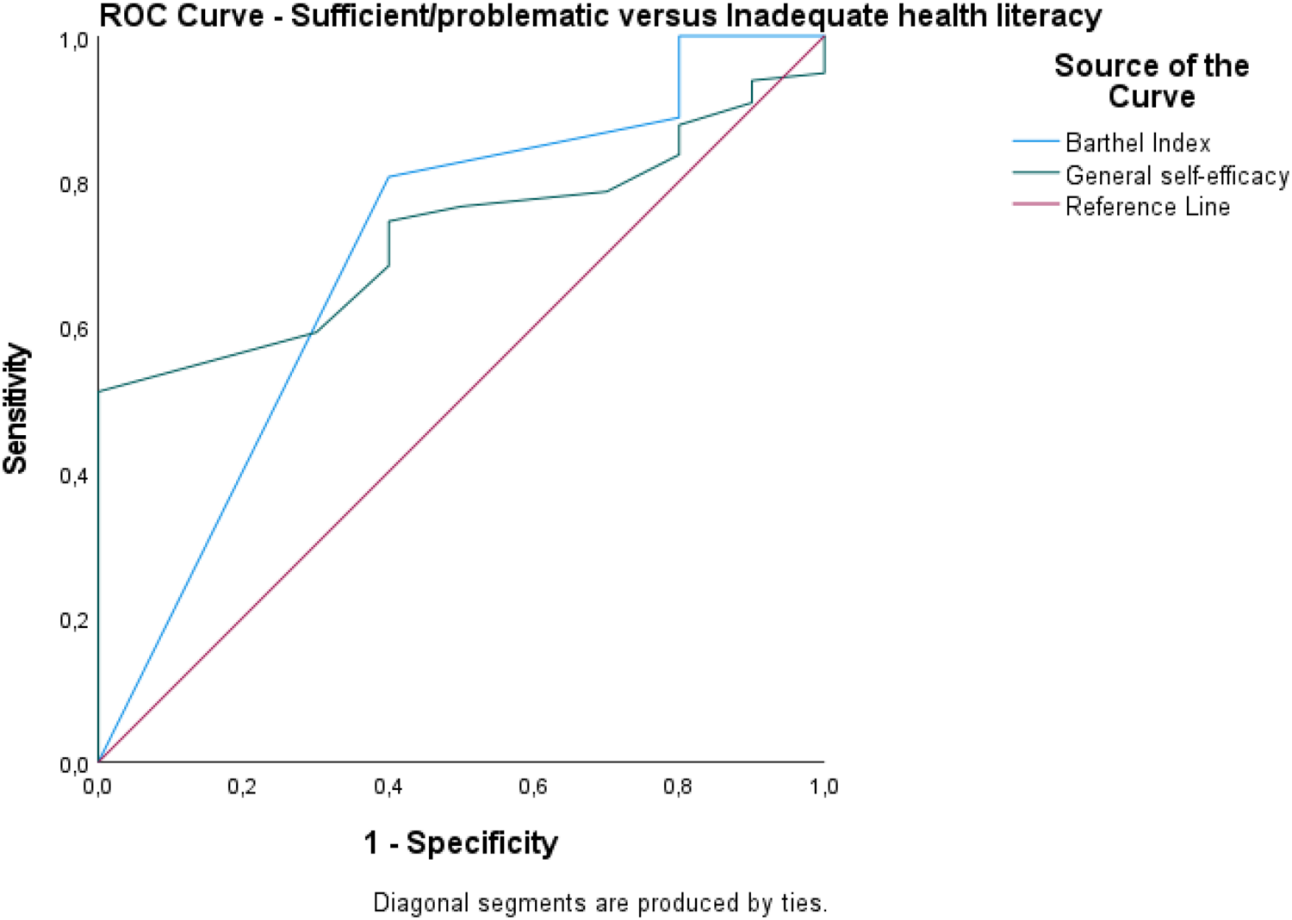
Specificity and sensitivity based on dichotomous variable health literacy (sufficient/problematic vs. inadequate).

The ROC curve for the predictions based on health literacy (sufficient/problematic vs. inadequate health literacy) is presented in Fig. 2. The area under the curve for general self-efficacy was 0.72 (CI 0.61–0.84), and for the BI it was 0.70 (CI 0.51–0.89). At a cut-off of 14.50 on the General Self-Efficacy Scale, the model presented 0.98 sensitivity and 1.0 specificity. At a cut-off of 70 on the BI, the model presented 1.0 sensitivity and 0.80 specificity.

## Discussion

This study investigated the association between general self-efficacy and health literacy among stroke survivors 1 year after discharge from hospital. General self-efficacy was positively associated with health literacy and with performing personal activities.

In this study, higher levels of general self-efficacy were associated with higher levels of health literacy in all statistical models. In other populations, an association between self-efficacy and health literacy has been identified for patients with diabetes^39,40^, patients with coronary disease^41^, patients in cardiac rehabilitation^42^, and older adults with hypertension^43^. In stroke survivors, a previous study found a positive association between health literacy and self-efficacy in the use and understanding of medication^44^. Therefore, it can be suggested that there is an interplay between general self-efficacy and health literacy, in which health literacy enables patients to understand and apply health information, whilst self-efficacy facilitates acting in the ways that are necessary to change health behaviors.

In addition, independence in performing personal activities of daily living was associated with higher levels of health literacy. This association has been less thoroughly studied^12^. It can be suggested that patients with greater health literacy are more liable to engage in behaviors that can enhance the performance of personal activities such as physical exercise^45^, but this association, as well as the impact of such proactive behaviors on stroke outcomes, needs further exploration.

In this study, the variables sex, age, education level, cognitive impairment, post-stroke fatigue, depression symptoms, and perceived impact on participation did not appear to be associated with health literacy in the adjusted statistical models. In contrast, one previous study found that lower levels of health literacy were associated with lower educational levels, lower income, multimorbidity, and/or moderate to severe functional limitations among patients with chronic diseases^46^. It is worth noting that social determinants and contextual factors such as healthcare organizations may play a role in health literacy, which may explain differences between the results of the different studies^47^.

The results of this study are relevant for understanding the interplay between general self-efficacy and health literacy. Previous programs for stroke self-management have found positive results regarding self-efficacy, indicating that our results could be used in future studies to tailor interventions that support both health literacy and general self-efficacy and could have the potential to improve the performance of personal activities^48^.

The literature addressing factors associated with health literacy among stroke survivors, especially regarding general self-efficacy, is limited. This study provides knowledge that could be used in the future for exploring pathways leading to improved health literacy among stroke survivors. Despite being a cross-sectional study with a small sample, the statistical models were built and analyzed with careful consideration of the relevant factors impacting upon recovery after stroke, based on the previous literature and clinical experience.

One limitation of this study concerns the population making up the sample. Most of the participants had experienced a mild stroke, which makes it difficult to generalize the results to survivors of a severe stroke or those with significant cognitive impairments. Furthermore, most of the sample presented a high level of health literacy and general self-efficacy. Therefore, the results may vary in a population with lower levels of health literacy and/or general self-efficacy 1 year post-discharge after a stroke.

## Conclusion

There is an association between health literacy and general self-efficacy, whereby stroke survivors with greater general self-efficacy also report higher levels of health literacy 1 year post-discharge from hospital. These results must be interpreted with caution due to the small sample size. Future studies should focus on the pathways by which health literacy and general self-efficacy work among stroke survivors and in populations with low levels of health literacy.

### Supplementary Information

Supplementary Information.

## Data Availability

The dataset generated during and/or analyzed during the current study are available from the corresponding author on reasonable request.
