# Supplementary material for: Association between general self-efficacy and health literacy among stroke survivors 1-year post-discharge: a cross-sectional study
Source: Sci Rep. 2024 Mar 27;14:7308. doi: 10.1038/s41598-024-57738-z (PMC10973423; doi:10.1038/s41598-024-57738-z)
Supplement: Supplementary file 1 — Supplementary Information. [file 41598_2024_57738_MOESM1_ESM.docx]

Supplementary information

*Legend: Area under the curve (inadequate/problematic versus sufficient).*

|  | | | | | |
| --- | --- | --- | --- | --- | --- |
| Test Result Variables | Area | Std. Error | Asymptotic Sig. | Asymptotic 95% Confidence Interval | |
|  |  |  |  | Lower Bound | Upper Bound |
| Barthel Index | 0.67 | 0.06 | 0.004 | 0.56 | 0.78 |
| General self-efficacy | 0.78 | 0.05 | 0.000 | 0.69 | 0.87 |

*Legend: Coordinates of the curve (inadequate/problematic versus sufficient).*

|  | | | |
| --- | --- | --- | --- |
| Test Result Variables | Positive if Greater Than or Equal To | Sensitivity | 1 - Specificity |
| Barthel Index | 29.00 | 1.00 | 1.00 |
|  | 47.50 | 1.00 | 0.98 |
|  | 70.00 | 1.00 | 0.95 |
|  | 77.50 | 0.90 | 0.93 |
|  | 82.50 | 0.99 | 0.88 |
|  | 87.50 | 0.96 | 0.83 |
|  | 92.50 | 0.94 | 0.78 |
|  | 97.50 | 0.90 | 0.56 |
|  | 101.00 | 0.00 | 0.00 |
| General self-efficacy | -1.00 | 1.00 | 1.00 |
|  | 6.00 | 1.00 | 0.98 |
|  | 14.50 | 1.00 | 0.95 |
|  | 17.50 | 0.99 | 0.95 |
|  | 18.50 | 0.99 | 0.90 |
|  | 19.50 | 0.99 | 0.85 |
|  | 20.50 | 0.99 | 0.81 |
|  | 21.50 | 0.97 | .81 |
|  | 23.00 | 0.96 | 0.73 |
|  | 24.50 | 0.96 | 0.68 |
|  | 25.50 | 0.93 | 0.68 |
|  | 26.50 | 0.88 | 0.61 |
|  | 27.50 | 0.88 | 0.51 |
|  | 28.50 | 0.85 | 0.49 |
|  | 29.50 | 0.79 | 0.44 |
|  | 30.50 | 0.72 | 0.32 |
|  | 31.50 | 0.64 | 0.17 |
|  | 32.50 | 0.61 | 0.15 |
|  | 33.50 | 0.52 | 0.10 |
|  | 34.50 | 0.39 | 0.10 |
|  | 35.50 | 0.31 | 0.10 |
|  | 36.50 | 0.24 | 0.07 |
|  | 37.50 | 0.16 | 0.05 |
|  | 39.00 | .119 | 0.00 |
|  | 41.00 | 0.00 | 0.00 |

*Legend: Area under the curve (sufficient/problematic versus inadequate).*

|  | | | | | |
| --- | --- | --- | --- | --- | --- |
| Test Result Variables | Area | Std. Error | Asymptotic Sig. | Asymptotic 95% Confidence Interval | |
|  |  |  |  | Lower Bound | Upper Bound |
| Barthel Index | 0.70 | 0.10 | 0.04 | 0.51 | 0.89 |
| General self-efficacy | 0.72 | 0.06 | 0.02 | 0.61 | 0.84 |

*Legend: Coordinates of the curve (sufficient/problematic versus inadequate).*

|  | | | |
| --- | --- | --- | --- |
| Test Result Variables | Positive if Greater Than or Equal To | Sensitivity | 1 - Specificity |
| Barthel Index | 29.00 | 1.00 | 1.00 |
|  | 47.50 | 1.00 | 0.90 |
|  | 70.00 | 1.00 | 0.80 |
|  | 77.50 | 0.98 | 0.80 |
|  | 82.50 | 0.96 | 0.80 |
|  | 87.50 | 0.92 | 0.80 |
|  | 92.50 | 0.89 | 0.80 |
|  | 97.50 | 0.81 | 0.40 |
|  | 101.00 | 0.00 | 0.00 |
| General self-efficacy | -1.00 | 1.00 | 1.00 |
|  | 6.00 | 0.99 | 1.00 |
|  | 14.50 | 0.98 | 1.00 |
|  | 17.50 | 0.97 | 1.00 |
|  | 18.50 | 0.95 | 1.00 |
|  | 19.50 | 0.94 | 0.90 |
|  | 20.50 | 0.92 | 0.90 |
|  | 21.50 | 0.91 | 0.90 |
|  | 23.00 | 0.88 | 0.80 |
|  | 24.50 | 0.86 | 0.80 |
|  | 25.50 | 0.84 | 0.80 |
|  | 26.50 | 0.79 | 0.70 |
|  | 27.50 | 0.77 | 0.50 |
|  | 28.50 | 0.75 | 0.40 |
|  | 29.50 | 0.68 | 0.40 |
|  | 30.50 | 0.59 | 0.30 |
|  | 31.50 | 0.51 | 0.00 |
|  | 32.50 | 0.48 | 0.00 |
|  | 33.50 | 0.40 | 0.00 |
|  | 34.50 | 0.31 | 0.00 |
|  | 35.50 | 0.26 | 0.00 |
|  | 36.50 | 0.19 | 0.00 |
|  | 37.50 | 0.13 | 0.00 |
|  | 39.00 | 0.08 | 0.00 |
|  | 41.00 | 0.00 | 0.00 |
